# COVID-19's Impact on China's Strategic Emerging Industries: An Observation of Policy Difficulties

**DOI:** 10.3389/fpubh.2021.778548

**Published:** 2022-01-04

**Authors:** Dexuan Li, Wensheng Dai, Weimin Guan

**Affiliations:** ^1^School of Economics, Peking University, Beijing, China; ^2^Financial School, China Financial Policy Research Center, International Monetary Institute, Renmin University of China, Beijing, China; ^3^School of Economics, Tianjin University of Commerce, Tianjin, China

**Keywords:** COVID-19 epidemic, R&D investment rate, financing constraints, baseline estimation test, PPML, Fourier Augmented Unit Root Test, Chinese strategic emerging industries

## Abstract

The study investigates the influence of the COVID-19 on the rate of R&D investment and foreign exchange development of China's most important emerging industry firms. From 2010 to 2020, data were collected from 26 locations across China, focusing on seven different types of critical creating companies. To analyze the data, we have applied Fourier Increased Unit Root Test, Granger causality assessments test, Pattern Assessment test, Poisson pseudo most excellent probability (PPML) approach, Wald test, and Regression analysis test. The results of the tests reveal a clear underlying association among COVID-19 relates Chinese exports and imports. COVID-19's instant effects on imports and exports lack working capital have been calculated, but the short-term, medium-to-long-term products are composite and unidentified. The article result main results are following: (i) The COVID-19 impacts the R&D investment is main industries like as high-end equipment industry, new materials industry, and new-era data innovation. (ii) The COVID-19 highly affects the imports and exports development network of Chinese strategic emerging industries which emphasizes cross-industry grouping features. The study provides the guidance to the future researchers to focus on COVID-19 affects on the strategic emerging industries of developed and underdeveloped countries to determine of foreign direct investment inflow and unemployment growth rates.

**JEL:** G20, O10, O40

## Introduction

The COVID-19 pandemic is affecting families, networks, and organizations worldwide; the episode of the COVID-19 pandemic is referred to as a “dark swan event” (1). It not only harms people's health around the world, but it has also harmed countries' financial activities. Controlling the epidemic has necessitated monstrous lockdowns and travel restrictions, which have changed human behaviors and hampered contemporary exercises, posing challenges and vulnerabilities to the global economy and human social systems (2, 3). Therefore, this study aims to investigate the effects of the COVID-19 on the rate of R&D investment and foreign exchange development of China's emerging industry firms.

By the beginning of April 2020, the repression policy has kept 81% of the global labor force at home (4), resulting in a 20% reduction in global assembly yield (5). In the meanwhile, the movement request was meeting with little success, with 90% of the world's air armadas grounded (6). COVID-19 has undoubtedly had a significant impact on the car industry, with production lines being shut down, supply networks being disrupted, and customer demand being reduced (7). We chose China for our evaluation since it was the first country to announce COVID-19 and are the best at mitigating the pandemic's effects. The blueprint for the 13th long-term plan for the People's Republic of China's public financial and social development advances the prerequisites and plans to improve the supporting role of emerging businesses, develop and foster key industries, build another example for the advancement of emerging ventures, and further develop the arising ventures' development climate (8).

This study aims to focus on how the intelligent Covid-19 resurgence in 2020 impacted China's central emerging industries. Wen et al. (9) study the impact of COVID-19 on China's economy to determine the yield of ventures, which results in a drop in capital consumption, experience, and a fall in utilization interest. Currently, China has one of the world's fastest-growing financial exchanges (10–12). Despite various studies demonstrating a substantial premium on its market, Chinese travel industry businesses have received insufficient attention (13). Accelerating the growth of critical emerging industries is strategic for changing China's financial development model, increasing the nation's global intensity, and creating a development-oriented country. Since the incident in China, China has been subjected to various restrictions imposed by other countries. As a result, China's economy has shut down, and the economy has been heavily impacted. In any event, this plague will significantly impact commerce, with China's imports and prices significantly reduced (14). Most areas rely on local electronic data science and education, ventures, and assets to increase logical and innovative examination on COVID-19 anticipation and control, supplement the inadequacies of pestilence. The board controls innovation and speeds up the creative work of new advancements, strategies, items, and hardware for pandemic avoidance and control to combat this scourge. Experts in logical and mechanical disciplines must conduct primary research on infection transmission properties and apply their findings in the battle against the new Covid-19 (15). The rapid spatial spread of the COVID-19 plague outbreak prompted the World Health Organization (WHO) to declare a pandemic resulting in line terminations and mass isolation. The review's primary goal is to look into the impact of COVID-19 on R&D venture rates, imports, and fares in seven key emerging industries: biotechnology, very high-quality hardware industry, new material industry, new energy industry, intelligent and new energy vehicle industry, energy preservation and ecological insurance industry, and computerized innovation. The advancement of China's sophisticated economy inflicts a turn of events and the growth of automated innovations such as artificial intelligence, big data, distributed computing, 5G, whipping, and item organization. There is also a dearth of sophisticated and astute applications in strategic areas such as competent medical care, intelligent training, astute transportation, driverless driving, astute urban communities, quick assembly, and astute houses (16). In the worst-case scenario, the financial growth rate will fall by 2–3 % in the first quarter and 5% in 2020. In comparison to SARS in 2003, COVID-19 proves to be a deadly virus (17, 18).

The main research question of this study is to explore the influence of the COVID-19 on the rate of R&D investment and foreign exchange development of China's most important emerging industry firms. To the novelty, this study mainly focuses on the COVID-19 impacts on the growth rate of R&D investment Chinese strategic emerging industries as since last 5 years Chinese government especially focused on the innovative technologies in these seven strategic emerging industries to compete with the production and quality standards of products. Secondly, we have also measured how much COVID-19 disturbed the imports and exports of these strategic emerging industries products in the world, especially USA and European countries. The results reveal that compared to other factors, COVID-19 plays a more significant role in reducing R&D investment and international trade rate of strategic emerging economies in China. The contributions of this study are following: (i) The COVID-19 impacts the R&D investment is main industries like as high-end equipment industry, new materials industry, and new-era data innovation. (ii) The COVID-19 highly affects the imports and exports development network of Chinese strategic emerging industries which emphasizes cross-industry grouping features. To the implication, this study sheds light on the effects of COVID-19 on China's related industries, which lay a solid foundation to analyse the COVID-19 is how to affect the R&D investment and foreign exchange development in emerging economies.

This paper is organized as follows. Section Literature Review presents a review of the research literature conducted on strategic emerging industries and COVID-19 epidemics. The research methodology and Research design is explained in section Methodology. Section Research Analysis and Results revealed the analysis the results of research methods such as Fourier Increased Unit Root Test, Granger causality assessments test, Poisson pseudo most excellent probability (PPML) method. Conclusion and implications with limitations and future research directions are discussed in section Conclusion.

## Literature Review

COVID-19 began to spread in December 2019 and was first detected in early January 2020. In China, it first appeared in mid-to-late January (19). Despite the many types of chaos and data difficulties, we must remember that COVID-19 is, first and foremost, a humane exam (20). According to the study, continued proliferation inside existing structures and localized transmission in new buildings will result in a 0.3 to 0.7 % drop in global GDP growth in 2020. To begin with, disasters and anthropogenic natural concerns (21) and their capacity to alter objectors' perception impact travel and the travel business on many scales. According to the World Travel Industry Association (WTO), travel industry appearances in China decreased by 1.2 to 694 million in 2003 (compared to a similar time in 2002), and hotel occupancy rates plummeted by 10% (More out of control). Despite the COVID-19 attack in key emerging businesses, China's exports increased by more than 20% in November 2020 compared to November 2019, driven by prices for PPE, customer hardware, other purchaser merchandise, and notably therapeutic goods. Along these lines, China's inconvenient new price-quality tests for personal protective equipment (PPE), particularly veils carried out by China's Public Clinical Items Organization (NMPA), significantly slowed commerce. China's Ministry of Commerce (MOFCOM) announced new capabilities for clinical inventory send-outs on March 30, 2020. As a result, buyers' earnings have decreased, prompting a further drop in interest rates. Automobile manufacturers, except a few, have all ceased produce in Europe and China (22). Strategicly, the families' wages have decreased due to the epidemic, but their expenditure on food has remained relatively constant compared to other goods. Force is strategic for pushing economic development, particularly in growing business sectors such as those affecting well-being, education, industry, and sustainable urban communities, and the possibilities are endless. COVID-19 impacts the region, notably by causing a drop in interest rates, the financial pressure, and disruptions in the force production network, which are particularly vulnerable to these developments. The Chinese Communist Party (CPC) and the government took swift steps to combat the epidemic, including massive scale isolation, travel restrictions, and the disengagement and observation of suspected patients. COVID-19 has been a major test of China's general health framework and administration restriction, highlighting the critical importance of working on significant pandemic avoidance and control frameworks alongside public general health framework crisis the board (23). First and foremost, governments must balance financial fluctuations and general well-being (24). In the long run, as a fundamental component of human resources, happiness is a key driver of financial outcomes. China's use of energy and natural resources is currently characterized by a low overall use level, a broad use mode, and a reverse use method. Financial progress is needlessly reliant on asset and energy investment, which results in massive waste and pollution (25). In 2020, global growth was expected to be−4.9 %, 1.9 % lower than the April 2020 World Financial Standpoint (WEO) forecast. Its recovery is expected to be more rapid than previously predicted (26). Despite its impact on economic mobility and energy consumption, the COVID-19 epidemic has made a significant contribution to environmental betterment, particularly in countries and domains that have implemented strict lockdown and constraint measures (27). The lockout implements a major travel day for those planning to return for the holidays one day before Chinese New Year's Eve. COVID-19 has a significant impact on China's transportation industry. Currently, the global energy usage of transportation sectors accounts for ~33% of total global energy utilization, with China accounting for around 20% of that (28). The mash and paper business encompasses a wide range of disciplines, including ranger service, agriculture, synthetic substances, science, conveyance, and transportation, and so plays an important role in the global economy (29). The COVID-19 pandemic has had a major impact on people's life, contemporary creation, and the global economy, which has unavoidably had a big impact on the mash and paper business. The government's sponsorship plan demands the electronic data industry's full and precise expansion, and it is also critical to its outcome. Combating the COVID-19 virus is also a test of the emerging online taxpayer-driven organization's development and social sustainability (30). The COVID-19 pestilence, triggered by the US-China trade war, had a significant impact on the Chinese economy and put it to the test (31). Because of the preponderance of highly developed financial business sectors in the United States, we use it as a benchmark in developing these rankings (32). Simultaneously, a new era of financial innovation known as Fin-Tech has emerged. A new industry of businesses employing online platforms, block chain, artificial intelligence, and other advancements to challenge conventional financial plans of action (33). Labor in the financial sector has worsened in many parts of the globe, not least due to the introduction of innovations, other business administrations, such as bookkeeping, corporate law, and business counseling, have experienced substantial growth (34). Fuchs et al. (35) used trade data in China to examine the exchange impacts of COVID-19. They investigate if historical financial ties established through trade and ventures and political ties are connected to China's fine example of strategic clinical items. In the first quarter of 2020, Hayakawa (36) investigated trade across 186 countries and discovered the COVID-19 issue has a significant negative impact on exchange trading nations, but not on bringing in the country. COVID-19's mission is to wreak havoc on hardware part suppliers by focusing on the prices of finished apparatus products. According to certain reports, a few clinics with somewhat weak financial foundations are in a dire financial situation in the medical care business (37). Local medical clinics have a lower working edge than city clinics, especially in the southern United States. Despite wearing heavy protective gear, there is a chance that clinical personnel will be too concerned about the risk of COVID-19 contamination (38). According to the (4), the COVID-19 impact on the world economy and has identified three possible scenarios for the unemployment rate of 188 million last year to between 5.3 million in their low-end scenario and 24.7 million in their very excellent quality situation, up from a base level of 188 million this year (4). The COVID-19 event has also impacted global oil prices and, by extension, financial growth (Gross domestic product). As a result, oil costs and financial development are frequently intertwined, and if both fall, there will be less abundant and fewer opportunities available in the labor market. It is critical to remember that oil prices were impacted not just by the Covid-19 event but also by subsequent events such as the OPEC meeting on oil production and the countries of oil producers and political tensions throughout the world. Despite the company's vulnerability to various shocks, its capacity, and capability to manage an emergency remain low in many ways. The tourist industry considers a significant transmission route that assists in the spread of numerous illnesses throughout the world owing to the mobility of affected people. The aviation sector was one of the most badly hit financial areas by COVID-19, according to Sun et al. (39), and it also had a significant role in spreading the virus early in the pandemic. According to Chung (40), the quick expansion of the aeronautics sector prepared for rapid multiplication. It spreads diseases throughout individual development, placing pressure on the world to take sufficient defensive measures to defend the global economy. COVID-19 was recognized as a global wellbeing problem of worldwide concern by the World Well-being Association on January 30, 2020 (41). As a result of this pattern, several countries reported a significant drop in power usage in commercial and contemporary areas (42), posing various challenges for electric utilities and system managers (43). The COVID-19 outbreak has impacted supply chains and drew the attention of analysts (44) and business experts all over the world. Furthermore, the pandemic's impact on the economy has been seen as a result of the worldwide lockdown of urban areas, work flexibility restrictions, travel boycotts, carrier suspensions seriously affecting the maintainability of supply chains in numerous organizations (45). The effective use of social capital is critical for combating environmental vulnerability, increasing intensity, advancing new product creation and mechanical development, and improving execution (46). These developments reveal China's position in the pandemic-related shock, as well as its role in global creation sharing. To be sure, pandemic-related disruptions to China's central role in global supply chains have sent shockwaves across industry hubs from one side of the globe (47). The issue of unshipped and inactive holders at Chinese ports resulted in compartment shortages at major ports worldwide. The scourge's impact on China's economy is vital (28) is a big concern, especially for countries shipping out novel foods cultivated on the ground. The travel industry is extremely vulnerable to pandemics, and the impact of regular risks on the retail industry with the conclusion that regular risks frequently result in a high retail shop conclusion proportion, as well as a decrease in the number of small retailers (48). The COVID-19 pandemic has substantially influenced corporate development and has had a considerable impact on popular life owing to widespread closures (49). To have a stable future beyond COVID-19, organizations should look for ways to improve the maintainable evolution of the store network (50). Research and development investment impedes financial skills development, which contradicts the previous conclusion that research and development investment constantly cultivates productive financing (51). Jiao (52) demonstrated that recognizing high mechanical levels need significant public funding for research and development ventures. Apart from the severe social and financial consequences of COVID-19 lockdown, the isolation guidelines resulted in a few ecological improvements in a few countries (53). According to figures provided by China's Public Agency of Measurements on April 1, 2020, China's GDP growth ratio fell by 6.8% in the first quarter of 2020 (54). During the climax of the country's COVID-19 lockdown in the spring of 2020, the Spring Celebration excursions caused a brief outflow dip akin to the odd effects of the COVID-19. Our month-to-month CO_2_ emissions estimates, based on daily data, are consistent with previous studies (55), which depicted the magnitude of the global month-to-month CO_2_ emissions decline in February 2019 due to decreased energy interest during China's Spring Festival. During the flare-up of the COVID-19 pandemic and the following local area lockdown to limit the spread, these benefits were mostly exploited by a few SMEs in high-level economies (56).

## Methodology

### Data Collection Process

The research will run from 2010 until 2020. Table 1 displays a list of 26 categorized business sectors drawn from seven distinct categories of key developing industries.

**Table 1:** Shows the 26 industries classified in eight strategic emerging industries.

**SR. NO**.	**Strategic Emerging Industries**	**Sector**	**SR. NO**.	**Strategic Emerging Industries**	**Sector**
		Chemical raw materials and products indust			
		Chemical fiber industry			
Case A	New material industry	Rubber and plastic products Non-metallic mineral products industry Ferrous metal smelting and rolling industry Non - ferrous metal smelting and rolling processing industry Metal products industry	Case D	New energy automobile industry	Automobile industry
		The production and supply of water	Case E	New energy industry	Power and heat production and supply industries
Case B	High-end equipment industry industry	Industry of railway, shipping, aerospace, and other transport equipment	Case F	Biological industry	Agricultural and sideline food processing
		Electrical machinery and equipment industry			
		Instrument industry			Food industry
		Metal products, machinery, and equipment repair industry Extracting oil and gas Mining of ferrous metal			
Case C	Energy conservation and environmental protection industry	Mining and cleaning of coal are two different industries.			Pharmaceutical industry
		The mining of nonferrous metals	Case G	The new generation of the information technology industry	Computers, communications, and other electronics
		Non-metallic mining industry			
		Petrochemical, coking, and nuclear fuel processing industries			
		General equipment industry			
		Special equipment industry			

*Source: List of these strategic emerging industries get from http://www.xinhuanet.com/*.

We gathered data from 450 companies, including 26 sectors from the seven types of strategic emerging industries (including bio and medicine, high-end equipment industry, new material, new energy, intelligent and new energy vehicle industry, energy conservation, environmental protection industry, and digital creative industry). COVID-19 data is obtained from the World Health Organization website (https://covid19.who.int/region/wpro/country/cn) and is utilized from December 01, 2019 to April 10, 2020. The CSMAR database provided information such as R&D investment statistics and financial indicators.

### Research Design

The fundamental idea behind creating the industry accounting index is as follows. To weight the equity and calculate the bookkeeping index *A*_*i*_*(t)* represents industry *i* and quarter *t* by analyzing each industry's periodical secretarial directory second *a*_*i*_*(t)*. Finally, create an optimal industry accounting index*, Yi*, using the synthetic index compilation approach (t). The cost index shows the industry's operational expenditures every quarter. The cost index comprises operating costs, taxes and levies, sales expenditures, and management charges. The price, leverage, and inventory indices are all negative. As a result, a drop in these indexes is advantageous to the industry's expansion. The final industrial accounting index *A*_*i*_*(t)* is obtained after passing through the standardization and compilation of synthetic indexes. The steps involved in creating a composite index are as follows.

Step 1: We utilize the method to decrease the impact of seasonal fluctuations and irregular Variables on the index computation *A*_*i*_(*t*) to calculate the symmetric change rate, use the current and prior periods as the base–so that the change is equal/symmetric whether the index increases or decreases. The symmetric change rate is calculated as follows:


$$C_{i}(t)=\;\{\begin{array}{c} \frac{A_{i}\left(t\right)-A_{i}\left(t-1\right)}{A_{i}\left(t\right)+A_{i}\left(t-1\right)}\;\times \;200,\;A_{i}\left(t\right)\;>0\\ A_{i}\left(t\right)+A_{i}\left(t-1\right),\;A_{i}\left(t\right)\;\leq 0\; \end{array},$$

The *C*_*i*_(t) described the rate of change, *A*_*i*_(t) represents the industry bookkeeping index, *i* represent industry, and *t* represents the quarter.

Step 2: To reduce the impact of excessive change in the index, we normalized the symmetric change rate. First, we determined the normalization factor *U*_*i*_(t):


$$U_{i}(t)=\;\frac{\sum_{t=2}^{n}\left|\;C_{i}(t\right)}{n-1}$$

The pace of change is enhanced to show the influence of COVID-19 on various industry indices more clearly while maintaining the index's stability. The standardized rate of change is a measure of how quickly something changes *R*_*i*_(t) is attained:


$$\begin{array}{l} R_{i}\left(t\right)=\;\frac{R_{i}\left(t\right)\;}{H_{i}}\;\times \;10000 \end{array}$$

Step 3: Calculate the composite index of each subsequent quarter, which is the final industry accounting index *Y*_*i*_(t), when *Y*_*i*_(t) = 100:


$$\begin{array}{l} Y_{i}\left(t\right)=\;Y_{i}\left(t-1\right)\times \;\frac{200+R_{i}\left(t\right)\;}{200\;-R_{i}\left(t\right)} \end{array}$$

### Imports and Exports Empirical Framework

This section outlines our experimental design for investigating COVID-19's impact on global imports and fares. We examine these impacts by looking at data on respective imports and exports month by month from December 2019 to April 2020. The previous section posits that COVID-19 damages in sending countries influence imports and fares *via* the evolution of import and fares expenditures and supply constraints. COVID-19 injuries in bringing in nations also affect imports and exports through changes in the cost of imports and exports and the rate of interest. The recurrence research of imports and exports esteems on specific nations' COVID-19 damages was not established to investigate the absolute influence of COVID-19 in bringing in Chinese trade. The following is how we came up with our pattern model:


(1) $$\begin{array}{l} Imports\text{ \& }Exports_{ijym}=\;exp\{a_{1}COVID_{iym}+\beta_{1}COVID_{jym}\\ \;+\delta_{ijy}+\delta_{ijm}+\delta_{ym}\}.\epsilon_{ijym} \end{array}$$

*Imports and exports*_*ijym*_ is the export value from countries *i* to *j* in month *m* year *y*. As explained in more detail later, *COVID*_*iym*_ and *COVID*_*jym*_ are the extent of COVID-19 damages in exporting and importing countries, respectively. We controlled for three kinds of fixed effects (δ_*ijy*_, δ_*ijm*_, and δ_*ym*_). _*ijt*_ is a disturbance term. We estimated this equation using the Poisson pseudo maximum likelihood (PPML) method. Then, we investigated the time-series changes of the coefficients for the COVID-19 variables. To this end, we estimated the following equation:


(2) $$\begin{array}{l} Imports\;\&\;Exports_{ijym}=\text{ exp}\{COVID_{iym}\;D\prime_{m}\alpha \\ \;+COVID_{iym}\;D\prime_{m}\beta +\delta_{ijy}+\delta_{ijm}+\delta_{ym}\}.\epsilon_{ijym} \end{array}$$

All coefficients in both the imports and exports are strategic and D is a vector factor indicating monthly loss occurred due to COVID-19 impacts on Chinese strategic emerging industries products. It also denotes the other confirmed cases, more passing, more inactivity in retail or the workplace, and more days with lockdown orders. We lead the Wald test of the coefficient for importers' COVID-19 effects are equal to that for Exporters' COVID-19 impacts to think about the greatness of the edge impacts.

### Fourier Augmented Unit Root Test

The Dickey-Fuller unit root test equation can be described as follows.


$$\begin{array}{l} Y_{t}=\beta_{t}+\sigma \;Y_{t-1}+\delta_{t}+\epsilon_{t}\;\left(i\right) \end{array}$$

Where β_*t*_ refers to a time-dependent function root is tested by examining, σ = 1 which shows a term that might lead to biased test results when the deterministic time is unknown.

### Granger Causality Estimations

Granger causality is a method for determining the causal relationship between two elements in a time series. The process is a probabilistic record of causality that uses observational informational collections to find examples of relationships. Even though it isn't the same, causality is strongly associated with conditions and logical outcomes. The invalid theory for the test is that slacked x-values don't clarify the variety in y. As such, it accepts that x(t) doesn't Granger-cause y(t). You may theoretically use the Granger Test to check if two elements are linked at a single instant on schedule. In any event, that version of the test is only used on rare occasions because it isn't beneficial, so I've left it out of this discussion.

#### Steps for the F-Test

The approach might get complicated due to many options available, such as browsing a variety of circumstances for f-esteem estimates. By using programming, you can bypass the majority of the intermediate strides. Some well-known financial affairs programming packages, such as E-Perspectives and PC-Give, rely on the Granger causality test to double-check that your time series is correct. We should change the Data to avoid the possibility of autocorrelation. You should also check for any unit roots in your model, as they can skew the test results. The following are the fundamental procedures for doing the test: the following two equations can be used to find f-value, if β_*i*_ = 0 for all lags *i*:


$$x(t)=\sum_{i=1}^{\infty }\beta_{i}x(t-i)+g_{i}+u_{i}(t)$$

$$x(t)=\sum_{i=1}^{\infty }\beta_{i}x(t-i)+\sum_{i=1}^{\infty }\alpha_{i}x(t-i)+g_{2}+u_{2}(t)$$

Two equations for Granger causality: Restricted (top) and unrestricted (bottom). Similarly, these equations test to see if *x(t)* Granger-causes *y(t)*:


$$y(t)=\sum_{i=1}^{\infty }\beta_{i}y(t-i)+g_{i}+\;\mu_{i}(t)$$

$$y(t)=\sum_{i=1}^{\infty }\beta_{i}y(t-i)+\sum_{i=1}^{\infty }\alpha_{i}y(t-i)+g_{2}+\;\mu_{2}(t)$$

Alternatively to calculate the f-statistic we can use the following equation:


$$\begin{array}{l} \text{F}=\frac{\left(ESS_{R\;}-\;ESS_{UR}\right)/q}{ESS_{UR}/\left(n-k\;\right)} \end{array}$$

## Research Analysis and Results

### Data Analysis Methods

To analyze the data, we have used the E-views version-10, and R-studio package my-data <- read.csv (“/shared/hartlaub@kenyon.edu/dataset_name.csv”) #use to read a csv file from my shared folder on R-Studio. For the data arrangement, Microsoft excel 2016 is used, and all the data is arranged in Panel data form.

### COVID-19 Short-Term Impacts

One of the short-term effects of the COVID-19 pandemic is reduction in the product of Chinese strategic emerging industries as items have been placed in retail outlets in April 2020 to satisfy the peak shopping season's demand (April through July). The scarcity of operating working capital during the COVID-19 crisis is the short-term consequence. This particular short-term effect has several secondary results. Companies are struggling to fund routine operational expenses, such as paying employees' salaries, covering the rent of the factory and warehouses, and paying energy bills, interest charges from bank loans, and other operating expenses, owing to reduced cash inflow. Furthermore, businesses are having difficulty obtaining a letter of credit (LC) to purchase source materials that will allow them to satisfy future demand. Given the present issue of product expiration, businesses are preparing to retain supplies on hand for the future and resume industry once the public-health limitations are lifted. Table 2 highlights the strategic emerging industries industry's short-term consequences of the COVID-19 pandemic, with an asterisk symbol indicating those firms recognized each impact.

**Table 2:** The short-term impact of the COVID-19 on the strategic emerging industries.

**Short-term impacts**	**Case A**	**Case B**	**Case C**	**Case D**	**Case E**	**Case F**	**Case G**	**Case H**
Loss as a result of a product's expiration (S1)	^*^	^*^	^*^	^*^	^*^	^*^	^*^	^*^
Working capital shortage (S2)	^*^	^*^	^*^		^*^	^*^	^*^	^*^
Expenses of routine operations are difficult to meet (S3)	^*^	^*^	^*^	^*^	^*^	^*^	^*^	^*^
The lack of cash causes a delay in the opening of LCs (S4)	^*^		^*^	^*^	^*^	^*^	^*^	^*^
Distributors and trade partners stop or curtail their activities (S5)	^*^	^*^			^*^	^*^	^*^	^*^

*Source: Authors*.

* *Indicates the result is significant*.

### Medium-to-Long-Term Effect

Effects our meetings revealed numerous medium-to-long-term impacts from the COVID-19 pandemic, in addition to the transient effects. One of the critical medium-to-long-term impacts mentioned by the respondents is a decrease in investment as a result. As a result, businesses may suffer the negative consequences of a lower return on original capital investment over the medium to long term (i.e., in the 3rd and 4th quarters of 2020). Table 3 summarizes the COVID-19 medium-to-long-term impacts on the Chinese strategic emerging industries. The mark image does represent the case organizations that referred to the separate effect.

**Table 3:** The medium-to-long-term impacts of the COVID-19 on Strategic emerging industries.

**Medium-to-long-term impacts**	**Case A**	**Case B**	**Case C**	**Case D**	**Case E**	**Case F**	**Case G**	**Case H**
Return on investment (ROI) reduction (L1)	^*^	^*^	^*^	^*^	^*^	^*^	^*^	^*^
In the industry, there are job cutbacks (L2)	^*^		^*^		^*^	^*^	^*^	^*^
Reduction of trade relationships (L3)	^*^	^*^			^*^	^*^	^*^	
The supply chain network is being rebuilt and restructured (L4)	^*^	^*^		^*^	^*^	^*^	^*^	^*^
The industry's contribution to GDP is being reduced (L5)	^*^		^*^	^*^	^*^		^*^	

*Source: Authors*.

* *Indicates the result is significant*.

Other medium-to-long-term effects include those affecting inventory network connections and design. A last medium-to-long-term contribution of the country total annual GDP growth, and there is possibility of a drop in popularity over the medium-to-long term will undoubtedly influence the strategic emerging industries obligations to the economy as a GDP growth participants.

### Fourier ADF Unit Root Test

Table 4 shows the Fourier ADF unit pulls tests in China separately, using a similar setup. Because of China, COVID-19 cases, deaths, imports and exports are all non-fixed at different levels. Even though the fares are level writing content, these characteristics have been the primary distinction.

**Table 4:** Fourier ADF unit root test.

	**Levels**	**1st variation**	**No. of Fourier**
COVID-19 cases	−2.689		1
COVID-19 deaths	−1.597	−3.198	1
Imports	−1.713	−5.088	1
Exports	−2.697		1

Note that the symbols ^***^, ^**^, and ^*^ represent the significance levels of 1, 5, and 10%, respectively.

*These estimates are from the author*.

There is no factor which sets at the following distinction the Fourier quantity is equal to 1. Such adversaries of exchange techniques hampered the smooth and successful flow of trade, such as COVID-19-related clinical equipment and other COVID-19-related healthcare equipments. Causality test results are presented in Table 5 which described the impacts of COVID-19 on Chinese international trade.

**Table 5:** COVID-19's influence on Chinese exports and imports: a causality test.

**Prob**.	**F-Statistic Adj R^**2**^**	***P***		
China				
Exports of COVID-19 Cases	2.4546	0.382	0.08	11
Exports of COVID-19 fatalities	4.897	0.09	0.07	11
Imports of COVID-19 Cases	1.107	0.08	0.06	11
Imports of COVID-19 fatalities	4.007	0.06	0.05	11

*The author discovers these estimations*.

Furthermore, enforced lockdowns severely impede the growth of unemployment, Covid-19 lockdown training and execution harmed the Chinese strategic emerging industries network since additional safety and security procedures increased exchange prices. However, in the long early stretches of 2020, China reduced veil fares and, on second thought, changed into a merchant.

### Parts and Component Trade Connections

Because China is the dominating start of imports in ultimate results, China is the key to solving this conundrum. Table 6 shows all items with the most significant import deficits from January, 2019 to April, 2020 amounting to more than 0.5% of overall imports from China for outcomes. Surprisingly, all items on this list are expected to be connected with both preceding cases: teleworking, sterilization, and remain at home/do-it-yourself in 2020 compared to 2019 (Do-It-Yourself).

**Table 6:** Measurements of Imports for Chinese strategic emerging industries.

**Products**	**Contribution to Gap 2020 (%)**	**Change in total imports, 2020%**	**Change in total imports from the world, 2020 (%)**	**Typical seasonality**	**DD (Gap2020–Gap2019)**	**Positive demand shock products**
Case A	35.3	4.6	2.86	Yes	Negative	
	9.3	1.21	0.75			TW
Case B	3.1	0.4	0.25			DI
	2.3	0.3	0.19			SH
Case C	2.2	0.29	0.18	Yes		
	1.7	0.22	0.14			SH
Case D	1.1	0.14	0.09			TW
	1	0.12	0.08	Yes		
Case E	0.8	0.1	0.06		Negative	
	0.7	0.1	0.06			SH?
Case F	0.7	0.09	0.06		Negative	
	0.6	0.08	0.05	Yes		
Case G	0.6	0.08	0.05		Negative	
	0.6	0.07	0.05			SH?

*The products listed here are those with contribution ratios of more than 0.5%. DD denotes differences between January 2020 and Gap2019, and TW, DI, and SH indicate teleworking disinfection and stay-home*.

*Source: Author's calculations*.

Teleworking-related products include PCs, tablets, information, and primary memory; imports of PC input/yield units have increased alarmingly since January 2020. Shower/power scattering devices, except agricultural and plant-based ones, should be used for sanitization. Since April, their imports have increased considerably. Coolers, hand-held force devices, and sanitizing contraptions may be goods that have increased interest from stay-at-home workouts.

### Baseline Estimation Results

To assess the severity of the COVID-19 damage, we used four methods. The first and second terms are the numbers of COVID-19 cases and deaths obtained from WHO's Sickness Prevention and Control Division reports. We increased the value of these integers by one and then collected their logs. Individuals' flexibility in terms of shopping and amusement and working settings is the third metric. We begin by sketching down the trade progressions. In a coordinated framework, Table 7 displays the month-to-month trades of Chinese strategic emerging businesses goods.

**Table 7:** Baseline estimation results.

	**(I)**	**(II)**	**(III)**	**(IV)**
Importers' COVID-19	−0.032	−0.023	−0.342	−0.083
Exporters' COVID-19	[0.003]	[0.004]	[0.052]	[0.026]
COVID-19 measure	Case	Death	Immobility	Lockdown
Wald statistics	0.049	0.478	4.087	0.02
Wald *P*-value	0.932	0.523	0.062	0.897
Log Pseudo likelihood	9.2+E 10	8.8+E 10	9.3+E 10	8.9+E 10
Pseudo *R*-squared	0.8789	0.9876	0.9356	0.9834
Number of observations	75,430	75,430	75,430	75,430

This table reports the estimation results using the PPML method. ^***^, ^**^, and ^*^ indicate the 1, 5, and 10% levels of statistical significance, respectively.

The errors in brackets are those that are grouped by country sets. We took into account country pair-year fixed impacts, country pair-month fixed impacts and year-month fixed impacts in all cases. All of the statistics in this table are based on data sent out. “Wald test” delves into the Wald test's application to the false hypothesis that the COVID-19 significance coefficient for importers and exporters is the same. “Wald p-value” is used to account for its p-value. The variable “COVID-19 evaluation” is used to assess the severity of the COVID-19 damage. The numbers of affirmed cases and passing are addressed separately under Case and Demise. The Lockdown variable also accounts for the number of days on which stay-at-home requests were feasible due to merchants' COVID-19 seriousness and the number of days on which work environment closing orders were successful due to exporters COVID-19 seriousness. The month-to month estimated trade values of Chinese strategic emerging industries are shown in the Table 8, which described these values in four columns.

**Table 8:** Monthly estimation results of Chinese strategic emerging industries trade values.

	**(I)**	**(II)**	**(III)**	**(IV)**
**Importers' COVID-19**				
1 for December	−0.023	−0.034		−0.632
1 for January	−0.042	−0.044		−0.227
1 for February	−0.027	−0.021	−0.398	−0.143
1 for March	−0.041	−0.025	−0.421	−0.315
1 for April	−0.032	−0.056	−0.378	−0.127
1 for May	−0.038	−0.026	−0.129	−0.053
1 for June	−0.022	−0.008	−0.054	−0.042
1 for July	−0.009	−0.003	−0.052	−0.49
1 for August	−0.007	−0.007	−0.111	−0.042
1 for September	−0.004	−0.014	−0.089	−0.093
1 for October	−0.005	−0.023	−0.075	−0.048
1 for November	−0.009	−0.009	−0.065	−0.031
1 for December	−0.003	−0.005	−0.121	−0.078
**COVID-19 Measure**	**Case**	**Death**	**Immobility**	**Lockdown**
Log pseudolikelihood	9.2+E 10	8.8+E 10	9.3+E 10	8.9+E 10
Pseudo R-squared	0.8789	0.9876	0.9356	0.9834
Number of observations	75,430	75,430	75,430	75,430

This table reports the estimation results using the PPML method. ^***^, ^**^, and ^*^ indicate the 1, 5, and 10% levels of statistical significance, respectively.

The findings of the evaluation using the PPML approach are presented in this table. ^***^ represents the 1, 5, and 10% degrees of factual relevance, ^**^, and ^*^, respectively. The standard errors are not responses; nevertheless, they were grouped by country pair for the study. We considered country pair-year fixed impacts, country pair-month fixed impacts, and year-month fixed impacts in every detail. All of the statistics in this table are based on trade data. The variable “COVID-19 measure” denotes the variable used to assess the severity of the COVID-19 damage. The numbers of affirmed cases and passing are addressed separately under Case and Passing. For shippers' COVID-19 seriousness, stability is defined as a % change in visits to retail and sporting outlets increased by an adverse one, and a comparable measure for exporters' COVID-19 seriousness is defined as visits to work settings. Similarly, the Lockdown variable tracks the number of days when stay-at-home requests were granted due to merchants' COVID-19 seriousness and the number of days when work environment shutdown orders were granted due to exporters' COVID-19 seriousness.

The results of the Table 8 shows that the Chinese exports surprisingly get reduction in a low level in the whole world during the first quarter of COVID-19 attack as the whole country was facing a non-stop lock down in the whole country. As a result of these circumstances by August 2020, transactions had returned to a similar level as the previous year. The standard errors are not replied to; instead, they were grouped by country pair for the examination.

### R and D Investment and Financing Constraints

The findings of the FIN and NAT relationship of words are shown in Table 9 and both FIN1^*^NAT and FIN2^*^NAT have negative and negligible coefficients. It indicates that financial development has minimal influence on decreasing R&D investment funding restrictions for state-owned and non-state-owned firms in strategic emerging industries.

**Table 9:** Shows the findings of the FIN-NAT relationship and R&D investment.

**RD**	**FIN–NAT interaction**	
Constant term	3.201^***^ (4.267)	4.081^***^ (4.998)
FIN1	0.201^***^ (3.741)	
FIN2		5.003^***^ (3.997)
FC	−0.092^***^ (−6.087)	−0.076^***^ (−6.023)
ROA	−1.306^***^ (−2.982)	−1.211^***^ (−4.031)
Growth	−0.201^***^ (−4.082)	−0.201^***^ (−4.007)
Cap	−0.312^***^ (−9.003)	−0.305^***^ (−9.051)
Q	0.108^***^ (7.005)	0.129^***^ (5.098)
Size	−0.194^***^ (−6.031)	−0.207^***^ (−6.003)
NAT	−0.003	−0.231^*^ (−1.783)
	(−0.041)	
FIN1^*^NAT	−0.208	
	(−2.091)	
FIN2^*^NAT		−2.722
		(−2.035)
Age	−0.011^*^ (−2.034)	−0.102^*^ (−2.072)
Year	Controlled	Controlled
Adjusted R squared	0.256	0.276
Prob>F	0	0

*** *Denotes a significant level of 0.01;*

* *denotes a significant level of 0.1*.

Table 10 shows the consequences of the FIN and SIZE connection period. At the 5% level, the FIN1^*^SIZE coefficient is negative and high, and FIN2^*^SIZE does not complete the significance evaluation. Compared to short and long foundation time ventures, intermediary financial advancement plays a more significant function in short foundation venture R&D expenditure. Long-term initiatives have long-term cooperation ties with banks, and their development is relatively steady, less influenced by funding requirements. The significance of financial exchange development isn't immediately apparent. Data transparency is highly valued in economic trade, and data variation is reduced to some extent. For companies with varying foundation timelines, there is no substantial difference in decreasing financing requirements.

**Table 10:** Shows the consequences of the FIN and SIZE connection time period.

**Research and development**	**FIN–SIZE relations**	
Constant items	2.081^**^ (4.087)	4.007^***^ (6.008)
FIN1	0.201^***^ (5.008)	
FIN2		5.038^***^ (3.098)
FC	−0.079^***^ (−6.002)	−0.092^***^ (−6.032)
ROA	−2.062^***^ (−5.002)	−2.005^***^ (−4.202)
Growth Rate	−0.214^***^ (−4.056)	−0.203^***^ (−4.081)
Cap	−0.224^***^ (−9.002)	−0.321^***^ (−9.034)
Q	0.098^***^ (7.078)	0.0792^***^ (6.009)
Size	−0.206^***^ (−6.003)	−0.206^***^ (−6.052)
NAT	−0.195^***^ (−4.087)	−0.197^***^ (−4.008)
Size	0.0076	−0.0087
	−0.943	(−1.543)
FIN1^*^SIZE	−0.202^***^ (−4.987)	
FIN2^*^SIZE		−0.846
		(−0.525)
Year	Controlled	Controlled
Adjusted R squared	0.308	0.266
Prob>F	0	0

*Note that*

*** *indicates that a level of 0.01 is significant*,

** *means that a group of 0.05 is strategic, and*

* *indicates that a level of 0.1 is necessary*.

The results in Table 10 did not affect the coefficients or meaning of other autonomous and control variables as explained in Table 9. Considering everything, the expansion of financial mediators and the securities market both play a more significant role in reducing financing limitations on strategic emerging industries R&D initiatives. Furthermore, based on financial events, there is no substantial difference between state-owned and non-state-claimed firms in terms of R&D funding requirements. Again, financial mediator advancement outperforms long-established ventures in terms of reducing financing demands for R&D investment. To be more precise, businesses of diverse sizes, types, and founding seasons should use various techniques to decrease data imbalance. The government should assist them in increasing R&D investment by entirely using the financial intermediary and securities exchange function.

## Conclusion

The master plan of this study is to learn the COVID-19's impact on Chinese strategic emerging industries using a significant information sway. This article examines COVID-19 impact on Chinese strategic emerging industries by making the link between financial events, financing constraints, and ventures' research and development supposition, to determine the growth rate of these industries international trade and R&D investment funds during the COVID-19 crisis period from December, 2019 to December, 2020. The study applied some research models to check the effects of COVID-19 on R&D investment and international trade. To analyze the data, we have applied Fourier Increased Unit Root Test, Granger causality assessments test, Pattern Assessment test, Poisson pseudo most excellent probability (PPML) approach, Wald test, and Regression analysis tests. The results reveal that compared to other factors, COVID-19 plays a more significant role in reducing R&D investment and international trade rate of strategic emerging economies in China. We used the original Fourier causality test to determine the causal linkages and the results revealed that COVID-19 has a negative relationship with R&D investment and international trade growth. The results also reveal that the COVID-19 pandemic's temporary impacts include more lapsed goods, a lack of working capital, difficulty in calculating functional expenses, a delay in opening LCs, and the termination of wholesalers' responsibilities such as financial constraints. It is also found that the short-term and medium-to-long effects of COVID-19 will most likely include a decrease in return on capital invested in strategic emerging industries. On the other hand, it has built negative relationships with importers, exporters, and a general reduction in the business' commitment to share their part in the annual GDP growth of China. Finally, a part of the short-term and medium-to-long-term consequences appear to be linked. Future research should explore these interrelationships since they may provide critical direction for developing good activity programs. Furthermore, the current review does not attempt to place the impacts and systems. Future analysis on the overall relevance of the approaches may help determine where businesses should focus their efforts first as they, along with the rest of the company; try to recover from the COVID-19 epidemic.

## Data Availability Statement

The original contributions presented in the study are included in the article/supplementary material, further inquiries can be directed to the corresponding author/s.

## Ethics Statement

Ethical review and approval was not required for the study on human participants in accordance with the local legislation and institutional requirements. The Ethics Committee waived the requirement of written informed consent for participation.

## Author Contributions

DL: conceptualization, methodology, data curation, supervision, and writing-reviewing and editing. WG: methodology, formal analysis, software, and investigation. WD: conceptualization and writing-reviewing and editing. All authors contributed to the article and approved the submitted version.

## Funding

This work was supported by major project of Beijing Social Science Foundation Research on Financial Support System Adapting to the Coordinated Development of Strategic Emerging Industries in Beijing-Tianjin-Hebei (Grant No. 20ZDA11).

## Conflict of Interest

The authors declare that the research was conducted in the absence of any commercial or financial relationships that could be construed as a potential conflict of interest.

## Publisher's Note

All claims expressed in this article are solely those of the authors and do not necessarily represent those of their affiliated organizations, or those of the publisher, the editors and the reviewers. Any product that may be evaluated in this article, or claim that may be made by its manufacturer, is not guaranteed or endorsed by the publisher.
